# A Comparative Double Blind Study of Nasal Dressing Sponge^®^ versus Merocel^®^ as Nasal Pack after Nasal Surgery

**DOI:** 10.22038/IJORL.2021.49606.2649

**Published:** 2021-11

**Authors:** Lorusso Francesco, Dispenza Francesco, Sireci Federico, Modica-Domenico Michele, Gallina Salvatore

**Affiliations:** 1 *Otolaryngology Unit, Department of Biomedicine and Advanced Diagnostic-University of Palermo, Palermo, Italy.*

**Keywords:** Merocel, Nasal pack, Epistaxis, Pain, Septoplasty

## Abstract

**Introduction:**

Nasal packing is a common procedure used to ensure haemostasis after nasal surgery.

**Materials and Methods:**

A prospective, randomized, controlled and double-blinded study was conducted on 80 consecutive subjects to investigate whether using Nasal Dressing Sponge® (NDS) instead of simple Merocel® might improve patients’ postoperative experience of nasal packing.

**Results:**

During the stay of the tampons no differences were noticed between the two groups as regards the postoperative pain. When it comes to pain during the packing removal, patients complained of worse symptoms in the side packed with Merocel. There was no bleeding after the removal of Merocel, whereas 5,6% patients were subject to some bleeding when NDS was removed.

**Conclusion:**

Merocel and NDS gave similar results regarding haemostatic activity. Nasal Dressing Sponge could decrease pain during the removal of the nasal pack, while it could be associated to a bigger incidence of mild bleeding after removing the pack.

## Introduction

Nasal packing is a common procedure used after endoscopic sinus surgery (ESS) and septoplasty to prevent postoperative complications (1-4). A nasal pack puts pressure between turbinate and septum preventing bleeding as well as the formation of synechiae and stabilizing the cartilaginous and bony framework (5). In septoplasty, it also supports the septal mucoperichondrial flaps and minimizes the risk of formation of septal hematomas (6).

However, despite its post-operative advantages, nasal packing is often remembered as a bad experience by patients mainly because of pain during the removal of packing. Through this procedure, the pack might cause trauma to the nasal mucosa resulting in altered mucuciliary clearance, bleeding, increased crusting, inflammation and synechia formation (7). Moreover, patients often complain of discomfort after the surgery because of the nasal obstruction, breathing disorders in sleep, oxidative stress, allergic reactions, dysphagia and eating difficulties (8). To improve the patients’ postoperative experience, different types of nasal packing have been proposed such as ribbon gauze with or without medication, absorbable biomaterials, telfa cellulose and foam, Merocel®, alginate, nasal splints, silastic sheets(8). At present, there is no consensus on the ideal material for nasal packing in the literature. The choice of nasal packing generally depends on the surgeon’s experience and the capacity to insert/remove the pack, the capacity to prevent bleeding and limit pain during the packing removal. Merocel® is a polyvinyl acetal compressed and dehydrated pack. It requires rehydration to activate it. To reduce pain during packing removal, Nasal Dressing Sponge ® was proposed. It might reduce formation of adherences and subsequently pain. However, a higher risk of bleeding is possible because of decreased haemostatic and absorbent capacities. In this study we have tried to investigate whether using Nasal Dressing Sponge® instead of simple Merocel® might improve patients’ postoperative experience of nasal packing in subject who underwent ESS and septoplasty.

## Materials and Methods

A prospective, randomized, controlled and double-blinded study was conducted by the department of Otorinolaringoiatria of the University of Palermo on 80 consecutive subjects (ranging from 19 to 61 years of age) who underwent endoscopic sinus surgery and/or septoplasty. The study protocol was explained to patients, and written informed consent was obtained from each subject. The study design was approved by the Human Research Ethics Committee of Palermo University.

Patients with a history of nasal surgery, sinonasal infections, sinonasal malignancy, bleeding disorders, anticoagulant therapy, any chronic comorbidity, or aged under 18 years or over 65 years were excluded.

All cases were performed by one surgeon. All the procedure was performed under general anesthesia with total intravenous anesthesia (TIVA).

Septoplasty was performed by means of a right hemitransfix incision and subperichondral dissection; all patients underwent radiofrequency decongestion of turbinate. The patient who underwent endoscopic sinus surgery (ESS), in 40 cases underwent bilateral medial antrostomy, anterior and posterior ethmoidectomy. A bilateral enlargement of the sphenoid sinus ostium was performed in the other 14 patients.

After surgery, the patients were subjected to nasal packing by an operator other than the surgeon: a random side was packedwith Merocel® and the other side with Nasal Dressing Sponge ®.All packings were removed 72h after surgery by one of the training assistant and again the patient was asked to make a mark on a visual analogue scale to represent the pain each pack caused on removal. Merocel**® **is a kind of foam pack made of polyvinyl acetal (PVA) and is packaged in a compressed, dehydrated state to allow ease of insertion. It requires rehydration with saline to activate it (9). Nasal dressing sponge**®**the dressing in mainly composed of PVA expanding sponge cover with a hemostatic gauze. When in contact with water, the gauze cover form a viscous gel and quickly stop capillary bleeding. In the meantime, the expanding sponge provides a controlled pressure on the bleeding site. Each patient underwent antibiotic therapy for six days after surgery. Patients were investigated in terms of bleeding and postoperatively pain at 1 hour, at 6 hours, at 1 day and during packing removal. A visual analog scale (VAS) ranging from 0 (no symptoms) to 10 (the most severe symptoms) was used to determine discomfort and pain. 

Bleeding during packing removal was graded as follows: 0, no bleeding; 1, mild bleeding (controlled spontaneously without any intervention); 2, moderate bleeding (controlled by the insertion of ephedrine-soaked cottonoids); and 3, severe bleeding (controlled by repacking or reintervention). The patients were followed up weekly for 4 weeks after surgery. At each follow-up visit, nasal endoscopy was performed to look for inflammation, crusting, adhesion and sinechiae. Statistical analysis was conducted using the Matlab® computer programme; χ2 test, odds ratio, and/or Fisher Exact test were used, following standard application conditions. Significance was set at 0.05.

## Results

Eighty subjects, 47 male and 33 females, ranging from 19 to 60 years of age (mean age = 42.97±12.28) were recruited. 54 patients suffered from chronic rhinosinusitis with nasal polyps (CRSwNP) and nasal septum deviation (NSD), 26 just from septal deviation and inferior turbinate hypertrophy. 26 patients (32,5%) underwent septoplasty, 54 patients (67,5%) underwent ESS and septoplasty. After surgery 56 patients underwent right nasal packing with Merocel and left nasal packingwith Nasal Dressing Sponge ® (NDS), while 24 patients underwent left nasal packing with Merocel and right with NDS. About the postoperative pain no difference between the two groups were noted at 1 hour, 6 hours and 1 day (P=0.1596; P=0.1783; P=0.1213).

Regarding pain during the packing removal, patients complain of the worse status of the side packed with Merocel (VAS 5.80 ±2.86) in comparison with the side packed with NDS (VAS 2.90 ±1.75) (P=0.0001) (Table 1).

After packing removal no bleeding was observed in patients treated with Merocel while 6 patients (5,6%) suffered from grade 1 bleeding when NDS was removed (P=0.0093). No grade 2 and 3 bleeding were observed.

An interesting fact is that, in general, a greater pain was shown during the removal of the swabs, respectively, both Merocel and NSD following septoplasty compared to those subjected to septoplasty and ESS. This could be explained in the light of a greater integrity of the mucosa and because of the pain perception during the removal of tampons in septoplasty.

**Table 1:** Pain during during the stay of the tampons and at removal. Visual analogue scale (VAS), standard deviation (SD)

	**Mean VAS**	**SD**	**P value**
1h pain			
Merocel	4.03	1.69	
NDS	3.43	1.57	0.1596
6h pain			
Merocel	3.47	1.87	
NDS	2.80	1.92	0.1783
1 day pain			
Merocel	2.70	1.97	
NDS	1.97	1.63	0.1213
Packing removal pain			
Merocel	5.80	2.86	
NDS	2.90	1.75	0.0001
			

## Discussion

Nasal packing is a procedure commonly used after ESS and septoplasty (1,2). It could prevent bleeding, the formation of synechiae and support the nasal structures reducing complications after surgery. However, this procedure is often associated with pain during nasal pack removal procedure, pressure, nasal obstruction, postnasal drip, dysphagia, and sleep disorders (10).

Postoperative pain is considered to be the most common morbidity associated with packings used in septoplasty. In addition, nasal pack may result in significant mucosal injury and loss of ciliary function. Many attempts, such as shortening the duration of packing and developing new packing material, have been made to minimize the morbidity associated with packing materials.

Some authors suggested that nasal pack could be avoided after septoplasty, but this protocol could be associated with more postoperative bleeding and subsequent formation of scars, synechiae, nasal obstruction and necessity of nasal packing (5,11,12).

Over the years, different types of nasal packing (e.g. hyaluronan, bovine gelatine mixed with thrombin, tissue adhesives, and biodegradable synthetic polyurethane foam) have been proposed to allow better patients’ experience after surgery (10). Research mainly focused on the reduction of nasal pain during pack removal and restoration of postoperative nasal homeostasis.

Unlike all the other studies, in this instance the patient himself is packed with two different types of tampon in each nasal fossa, so, this dispels the bias related to the different perception of pain in individual patients.

 Merocel is a kind of foam pack made of polyvinyl acetal and is packaged in a compressed, dehydrated state to allow ease of insertion. It requires rehydration to activate it. 

The pores of the PVA swabs swell, causing hemostasis thanks to the retention of the clot and the pressure exerted on the walls of the nasal pits. However, the most important disadvantage of simple Merocel® is pain. This occurs during the insertion of the swab, if the patient is awake while the insertion in being carried out, during its stay in the nasal fossa and particularly, during the removal (13-14).

Many studies have evidenced that when plain Merocel® in used, it adheres to the bleeding site, the incision site, and other raw areas over the septum. During its removal, the pack dislodges from the site of adherents, causing trauma to the nasal mucosa, which results in altered mucociliary clearance, bleeding, increased crusting, inflammation, and synechia formation (13,14).

According to some authors, these disadvantages may be overcome by using finger‐gloved Merocel® instead of simple Merocel® (13,14).

In our opinion, the simple finger of a glove to cover Merocel reduces some of these disadvantages related to adherence to the walls of the nasal cavities but, more significantly, it reduces the haemostatic and absorbent power of Merocel.

For this reason, we decided to compare Merocel with a pad covered with a grid of "haemostatic material" which does not compromise its absorption characteristics. At the same time, it facilitates he removal procedures while safeguarding the integrity of the mucosa.

In a study of Kaur et al,. 60 patients underwent nasal packing with gloved or ungloved Merocel after septoplasty. They reported less pain with gloved Merocel during pack insertion and removal and early normalization of saccharin transit time (STT) (7). On a prospective study of 37 patients who underwent ESS, gloved Merocel caused less discomfort during removal than ungloved Merocel while no differences were evidenced on their effects on sinonasal mucosal inflammation and postoperative discomfort (15).

In a study of 48 patients who underwent to ESS, gloved Merocel was superior to Silastic in terms of pain during the removal while no differences were noticed as to the incidence of synechiae and scarring and facial pain prior to removal and extent of discharge (16). Romano et al reported less pain during pack removal and less bleeding with Biodegradable Nasopore in comparison to ungloved Merocel (10).

In a study of 60 patients Saedi et coll (17) compare the effects of routine nasal packing with polyvinyl acetal sponge (Merocel) versus no packing, after endoscopic sinus surgery for nasal polyposis. This study found no significant difference between polyvinyl acetal packed and non-packed groups and supported the reconsideration of routine post-operative packing in selected cases.We did not highlight any significant differences in post-operative bleeding either during the stay or on the removal of the packs. We did not highlight a significant difference in pain between the two different types of packs. But we noticed a significant reduction in pain when removing the pack from the coated side. In fact, the haemostatic coating of the pack not only gives a greater haemostasis in contact with liquids but also creates a gelatinous substance which allows a much easier and bloodless removal of the pack. Instead, we found five episodes of displacement of the coated pack, two posteriorly and one anteriorly this due to the lesser friction inside the nasal cavity caused by the gel that is formed when the pad lining comes into contact with liquids, this dislocation can occur anteriorly usually due to a sneeze or posteriorly in the case of deep inhalations. The aspiration of the pack was prevented by the front binding of the swabs’ threads anterior to the columella. In the cases of posterior dislocation, the coated pack was repositioned by pulling on the anchor wire. In cases of anterior expulsion not being followed by epistaxis, the nasal cavity was not re-packed.

A greater formation of crusts and synechiae was also found in the first cases of the study from the side buffered with coated pad. In fact, it was noticed that once the swab was extracted, the gelatinous coating remained at the level of the nasal fossa going to dehydration and promoting a greater formation of crusts and therefore of synechiae. We saw that by providing the suction of the residual gelatinous material at the level of the nasal fossa at the end of the removal, the formation of crusts and synechiae was significantly reduced with values superimposable to those evidenced in the use of Merocel.

## Conclusion

The current study was conducted to evaluate postoperative outcomes such as bleeding and pain in patients who underwent nasal packing Merocel or NDS after ESS and septoplasty. Our results suggest that Merocel and NDS had similar results regarding haemostatic activity. Nasal Dressing Sponge could decrease pain during the removal of the nasal pack, while it could be associated to a bigger incidence of mild bleeding after pack removal. Further studies are necessary to understand which type of protocol should be used after nasal surgery.
